# Is the stability of ACTH in whole blood a genuine concern during the preanalytical phase? A systematic review

**DOI:** 10.11613/BM.2025.010502

**Published:** 2025-02-15

**Authors:** Ahmad Dib, Damien Leleu, Stéphanie Lemaire, Laurence Duvillard, Louise Ménégaut, Damien Denimal

**Affiliations:** 1 Department of Clinical Biochemistry, University Hospital of Dijon Burgundy, Dijon, France; 2 Center for Translational and Molecular Medicine, Unit 1231, Université Bourgogne Europe, Dijon, France

**Keywords:** ACTH, biochemistry, endocrinology, extra-analytical phase

## Abstract

Adrenocorticotropic hormone (ACTH) has historically been considered an unstable hormone after venous sampling, necessitating stringent conditions for the transport of blood samples to the laboratory to ensure accurate measurement. However, recent investigations suggest that ACTH may be more stable than previously assumed, raising the possibility of more flexible handling conditions. This prompted us to conduct a systematic review using the MEDLINE database to ascertain the stability of ACTH in blood samples. We included 9 studies in our final analysis from 405 reports. Our findings reveal that all studies reported a mean percentage difference (PD%) in ACTH concentrations relative to baseline below the 10% threshold when uncentrifuged tubes were stored under refrigerated conditions for 2, 4, 6, and 8 hours. In contrast, the mean PD% exceed the 10% threshold in 5 out of 7 studies investigating a storage duration of 24 hours under refrigerated conditions. Nearly all studies reported a mean PD% in ACTH concentrations relative to baseline below 10% when uncentrifuged tubes were stored at room temperature for 2, 4, and 6 hours. However, for storage durations of 8, 12, and 24 hours at room temperature, most studies observed a mean PD% exceeding 10%. In summary, our findings suggest that ACTH remains stable in uncentrifuged tubes containing EDTA for 6 h at room temperature and at least 8 h under refrigerated conditions. Our findings can assist clinical laboratories in reviewing their acceptance criteria for sample transport regarding time and temperature.

## Graphical abstract

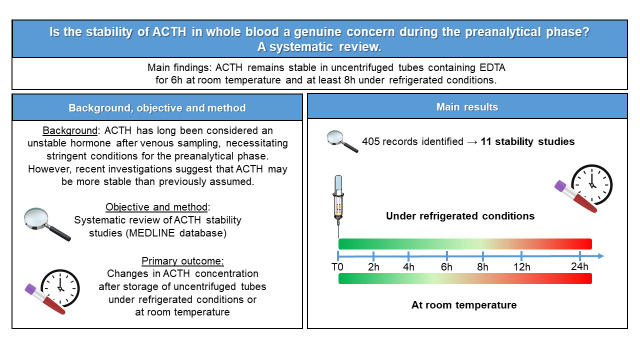

## Highlights

• Adrenocorticotropic hormone (ACTH) remains stable in uncentrifuged EDTA tubes for 6 h at room temperature

• ACTH is stable for at least 8 h in EDTA tubes kept under refrigerated conditions

• Handling conditions that are less stringent than those typically required by clinical laboratories are permitted

## Introduction

Human adrenocorticotropic hormone (ACTH) is a 39-amino acid peptide hormone produced by cells of the anterior pituitary gland. It acts as the primary stimulant of glucocorticoid synthesis and secretion by the adrenal cortex. Accurate measurement of circulating ACTH concentrations is pivotal for diagnosing disorders related to the hypothalamic-pituitary-adrenal axis, such as adrenal insufficiency and Cushing’s syndrome (*1*, *2*).

The preanalytical phase is particularly critical for ACTH analysis. In fact, it has long been suggested that ACTH may be unstable in whole blood after venous sampling, especially because of its degradation by blood cell proteases (*3*-*5*). Controlling the time and temperature of blood specimens before the centrifugation step therefore looks crucial to mitigate ACTH degradation. In fact, clinical laboratories often advocate for stringent conditions regarding the duration and temperature of transport to the laboratory of blood samples. For instance, a review of the preanalytical instructions issued by 33 university hospitals and private clinical laboratories across France revealed that 76% of them request transport under refrigerated conditions, while the remaining 24% accept transport at room temperature (RT) (personal unpublished review based on laboratory websites, conducted on March 7, 2024). The recommended maximum duration of transport of blood samples is heterogeneous, with 12% of laboratories suggesting a maximum of 30 min, 15% advising up to 2 h, 36% extending the limit to 4 h, while only 37% have acceptance criteria greater than 4 h.

In contrast to these current practices, recent published data suggest that ACTH may exhibit more stability in whole blood than previously assumed. In particular, two studies recently demonstrated that the storage of whole blood for 8 or 12 h at 4 °C did not impact plasma ACTH concentrations (*6*, *7*). In addition, Dong *et al.* recently found that ACTH was also stable in whole blood for 6 h at 22 °C (*7*). These recent findings raise questions about current practices in clinical laboratories, and this prompted us to conduct a systematic literature review to elucidate the stability of ACTH concentration in whole blood regarding storage duration and temperature. Such approach may contribute to the harmonization of preanalytical instructions across clinical laboratories (*8*).

## Materials and methods

The protocol for this systematic review was registered in the PROSPERO database (#CRD42024540452), and is presented according to the Preferred Reporting Items for Systematic Reviews and Meta-Analyses (PRISMA) 2020 statement (*9*).

### Eligibility criteria

Table 1 shows the study characteristics used to decide whether a study was eligible for inclusion in the review, according to the Population, Intervention, Comparison, Outcome, Study (PICOS) search question format.

**Table 1:** Eligibility criteria for the selection of studies

**PICOS parameter**	**Inclusion criteria**	**Exclusion criteria**
Population	Human blood samples from individuals of any age, gender, ethnicity, health conditions, and ACTH concentrations. Any collection tubes with any additives. Any analytical methods.	Non-human blood. Biological matrices other than blood (*e.g*., quality control materials).
Intervention	Short-term storage of whole blood (≤ 24 h) at a temperature higher than 0 °C before centrifugation.	Immediate centrifugation after blood collection (*i.e*., studies with storage of serum or plasma). Long-term storage (> 24 h) and/or at a negative temperature.
Comparison	Circulating ACTH concentrations at baseline (immediately centrifuged and analyzed or frozen after venipuncture).	
Outcomes	The primary outcome was the difference in ACTH concentration after storage (expressed in percentage difference from baseline). Any measure of the precision of the percentage difference in ACTH concentrations was collected (*e.*g., standard deviation or interquartile range).	Reports were excluded if the calculation of the percentage difference (PD%) in ACTH concentrations following storage was not directly available in the article.
Study design	Articles published in peer-reviewed journals. Any date of publication.	Articles not published in English, case reports, meta-analysis, reviews, expert opinions, conference reports.
ACTH - adrenocorticotropic hormone.		

### Information sources and search strategy

The search procedure was conducted using the PubMed/MEDLINE database. The search strategy was as follows: ((ACTH[Title/Abstract]) OR (adrenocorticotrop*[Title/Abstract])) AND ((stability[Title/Abstract]) OR (storage[Title/Abstract])). In addition, the references cited in the included studies were examined to select additional reports for screening. All reports published up to April 1, 2024, were included for further screening.

### Selection and data collection process

Two authors independently screened the titles and abstracts, excluding reports and studies based on the predefined inclusion and exclusion criteria. The remaining studies were integrally read to definitively judge on their inclusion. Disagreements on the eligibility of studies were resolved through discussion and consensus between authors. Data from each report were independently collected in Excel sheets by two authors. No automation tool was used in the selection process or in data collection.

### Data items (outcomes)

The primary outcome was the difference between the baseline concentration of circulating ACTH and the concentration after storage of whole blood in each condition, expressed as percentage difference (PD%). Data on the precision of the PD% in concentrations were also collected.

### Study risk of bias assessment

The risk of bias in the included studies was independently evaluated by two authors using an adapted version of the quality grading tool developed by Gomez-Rioja *et al.* for stability studies (*10*). Two criteria were omitted from the original quality grading tool as they were deemed inappropriate for our study: total drawn volume and time before centrifuging. Regarding the criterion “type of centrifugation”, when the authors did not specify adherence to the tube manufacturer’s instructions, we then considered as recommended the following centrifugation conditions: ≤ 1300xg for 10 min for tubes from Becton Dickinson (Franklin Lakes, USA), 1800-2200xg for 10-15 min for Vacuette tubes from Greiner Bio-One (Kremsmünster, Austria), and 2000xg for 15 min or 2500xg for 10 min for tubes from Sarstedt (Nümbrecht, Deutschland) (*11*-*13*).

The maximum score of the adapted version of the quality grading tool was 25 points (*10*). Studies were classified into four quality grades: excellent if the total score exceeded 18.75 points, acceptable between 12.5 and 18.75 points, doubtful between 6.25 and 12.5 points, and unacceptable if the score was lower than 6.25 points (*10*). Any discrepancies in judgement between the two authors were resolved through discussion.

### Effect measures

The effect measure of the primary outcome was the relative difference, expressed as PD%, between the baseline concentration of circulating ACTH and the concentration for each storage condition of uncentrifuged tubes.

Data on the precision of PD% values were expressed as standard deviation (SD). In cases where SD was not directly reported in the studies, it was calculated from other measures such as standard error mean, interquartile range and median, or error bars in graphs. Standard error mean was converted to SD using the following formula: SD = standard error mean x √n, where n represents the sample size. The approach outlined by Shi *et al.* was followed when SD was estimated from interquartile range, median and mean (*14*).

Establishing the maximum permissible difference indicative of a significant change in PD% is challenging in the case of ACTH. Such limit is typically determined from the total or acceptable change limits according to ISO 5725-6, based on analytical and within-subject biological variation (*15*). Unfortunately, biological variation data for ACTH were not available in either the EFLM biological variation database or the Ricos’ database (*16*, *17*). Therefore, we chose to adopt a threshold of 10% as maximum permissible difference for the mean PD%. This 10% threshold was widely used in previous studies focusing on ACTH stability (*6*, *7*, *18*-*22*).

### Synthesis methods

Data were classified into two groups according to whether they were from ACTH stability studies at RT or under refrigerated conditions. For each temperature condition, they were subsequently grouped according to duration storage. Since the degradation of ACTH is time-dependent, data from the single study examining 3 h storage duration was grouped into the “2-h” subgroup (*7*).

Forest plots were used to visually display results of individual studies and syntheses, and they were generated using GraphPad Prism (version 9.5.0) (GraphPad Software, Boston, USA).

## Results

### Study selection

**Figure 1:**
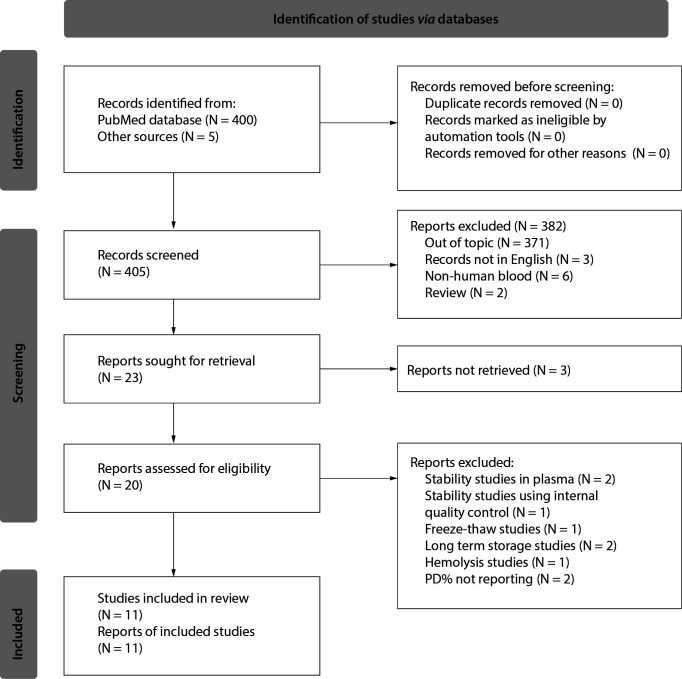
PRISMA 2020 flow diagram

The PRISMA flowchart depicted in Figure 1 illustrates the selection of studies. A total of 405 reports were identified through our systematic search strategy. The vast majority (N = 382, 94%) were excluded based on the initial screening of their titles and abstracts due to their lack of adherence to our inclusion criteria.

Among the studies assessed for eligibility (N = 20), two were excluded as they examined the stability of ACTH concentration in plasma after immediate centrifugation following blood collection, but not in uncentrifuged tubes (*23*, *24*). Two additional studies were excluded because they specifically investigated long-term plasma storage at - 20 and - 70 °C (*4*, *25*). Some other studies were eliminated due to their focus on the stability of ACTH using internal quality control rather than freshly collected blood samples, the effects of repeated freezing and thawing of plasma, or the impact of hemolysis on ACTH stability (*26*-*28*). Lastly, two studies were excluded because PD% was not directly reported in the original articles, and only mean concentrations were provided (*29*, *30*).

Ultimately, our systematic review included 11 studies, with almost half of them (N = 6, 55%) being published within the last decade. Table 2 summarized the main characteristics of these studies. In total, 151 blood samples were analyzed. Among the 11 included studies, ten studies provided stability data under refrigerated conditions, with 9 studies indicating a storage temperature of + 4 °C and one study reporting storage in ice/water. Additionally, nine studies presented stability data at RT, with two studies not specifying the exact temperature and the remaining studies reporting temperatures ranging from 22 to 25 °C. The RT condition in the study by Bosse and Bayard was not analyzed, as the collection tubes were actually kept in a cooling container (*6*). However, this study was retained because it also included a storage condition at 4 °C in a refrigerator, which fully met our inclusion criteria.

**Table 2:** Characteristics of the selected studies

**Study**	**Sample size**	**Collection** **tube**	**Time of storage**	**Temperature of storage**	**Analyzer** **/ method**	**Baseline ACTH concentrations**	**Reference**
Dong, 2024	3	EDTA-K_2_*	3, 6, 12 and 24 h	4, 22 °C	Immulite 2000 XPi (Siemens)	N.R.	(*7*)
Bosse, 2024	15	EDTA-K_2_ (Greiner)	1, 2, 4, and 8 h	4 °C and Crioplast at 22 °C	Liaison (Diasorin)	17.6 ± 7.4 pg/mL	(*6*)
Nandakumar, 2020	19	EDTA-K_2_ (Becton Dickinson)	2 and 4 h	4 °C	Elecsys Cobas 6000 (Roche)	12 samples: < 20 pg/mL 5 samples: 20-40 pg/mL 2 samples: > 40 pg/mL	(*37*)
Chakera, 2017	15	EDTA*	1, 6, 24 and 48 h	4, 20 °C	Modular E170 (Roche)	Median (range): 18.9 (7.7-759.6) pg/mL	(*22*)
Wu, 2017	21	EDTA-K_2_*	2, 4, 8, and 24 h	4, 22 °C	Modular E602 (Roche)	7.2-63.3 pg/mL	(*18*)
Christensen, 2016	30	EDTA-K_2_*	2, 4, 24, and 48 h	RT, ice/water	Cobas e601 (Roche)	28 samples within the reference range (7.4-63 pg/mL). 1 sample: 6.0 pg/mL	(*20*)
Oddoze, 2012	10	EDTA-K_3_ (Becton Dickinson)	4, 8, 12, 16 and 24 h	4, 25 °C	Cobas e601 (Roche)	N.R.	(*38*)
Reisch, 2007	19	EDTA (Sarstedt)	1, 2, 4, 24 and 48 h	4, 22 °C	Advantage (Nichols)	5-774 pg/mL	(*21*)
Ellis, 2003	10	EDTA*	0.5, 6, and 24 h	4, 24 °C	Radio-immunoassay (Nichols)	7.3-45 pg/mL	(*19*)
Diver, 1994	6	Lithium heparin*	6, 24, 48, and 168 h	4 °C, RT	Allegro HS (Nichols)	92-94 pg/mL	(*32*)
Lambert, 1985	3	Heparin*	1, 2 and 4 h	22 °C	Radio-immunoassay	152-212 pg/mL	(*31*)
*Manufacturer not provided. ACTH - adrenocorticotropic hormone. EDTA - ethylenediaminetetraacetic acid. N.R. - not reported. RT - room temperature.							

Ethylenediaminetetraacetic acid (EDTA) tubes were used in 9 of the 11 selected studies, while heparinized tubes were used in two studies.

ACTH measurements were performed using automated methods from Roche (Basel, Switzerland) in 5 of the 11 selected studies, Nichols (San Juan Capistrano, USA) in two studies, Siemens (Erlangen, Deutschland) in one study, and Diasorin (Saluggia, Italy) in one study, and using manual radioimmunoassays in two studies.

### Risk of bias

Table S1 (see Supplementary material) presented the assessment of risk of bias in the 11 selected studies. The quality grade was deemed acceptable for 6 of them, doubtful for 3, and unacceptable for the remaining 2 studies. The two studies deemed to be of unacceptable quality were excluded from the analysis.

### ACTH stability under refrigerated conditions

**Figure 2:**
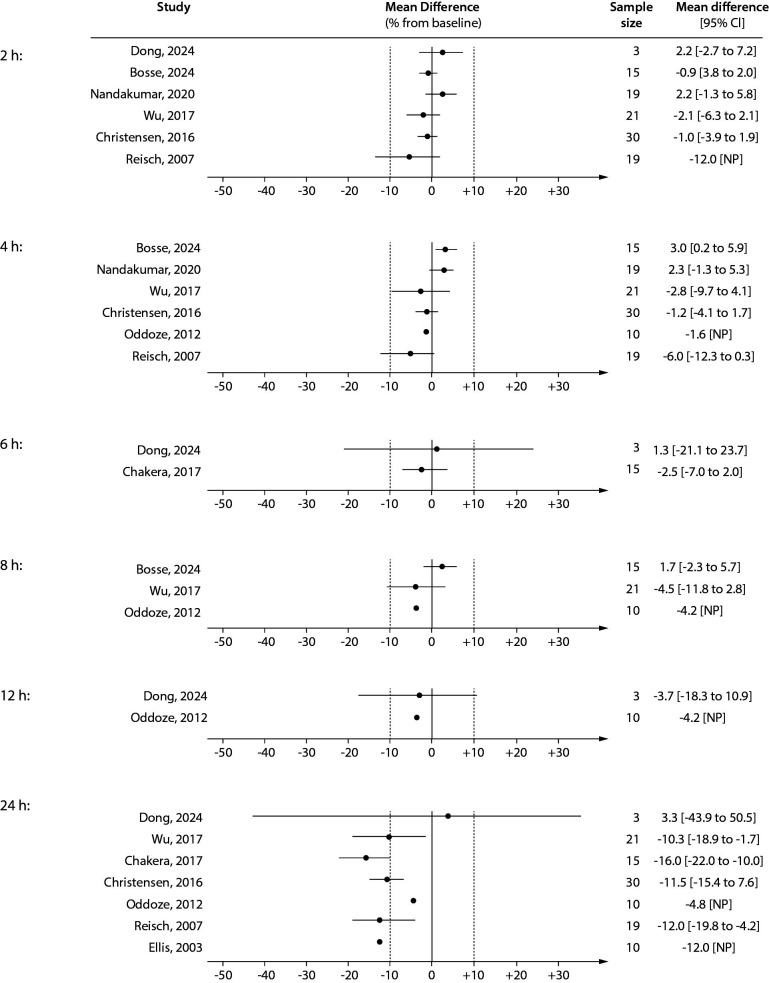
Forest plots depicting the stability of ACTH in uncentrifuged tubes under refrigerated conditions relative to the duration of storage prior to centrifugation. The X-axis represents the mean percentage difference (PD%) compared to the baseline concentration. Bar errors correspond to 95% confidence intervals (CI). ACTH - adrenocorticotropic hormone. NP - not provided (indicating the individual studies where the calculation of the 95% CI was not feasible with the available data).

Six studies provided data on the stability of ACTH concentration in uncentrifuged tubes stored under refrigerated conditions for a storage duration of 2 and 4 h, 2 studies for 6 h, 3 studies for 8 h, 2 studies for 12 h, and 7 studies for 24 h. All of these studies utilized collection tubes containing only EDTA. As shown in Figure 2, all stability studies reported a mean PD% in ACTH concentrations relative to baseline that remained below the 10% threshold when uncentrifuged tubes were stored under refrigerated conditions for 2, 4, 6, and 8 h. In contrast, in 5 out of 7 studies examining a storage duration of 24 h, the mean PD% exceeded the 10% threshold, with values ranging from - 16.0% (95% confidence interval (CI): - 22.0 to - 10.0) to + 3.3% (95% CI: - 43.9 to 50.5).

### ACTH stability at room temperature

**Figure 3:**
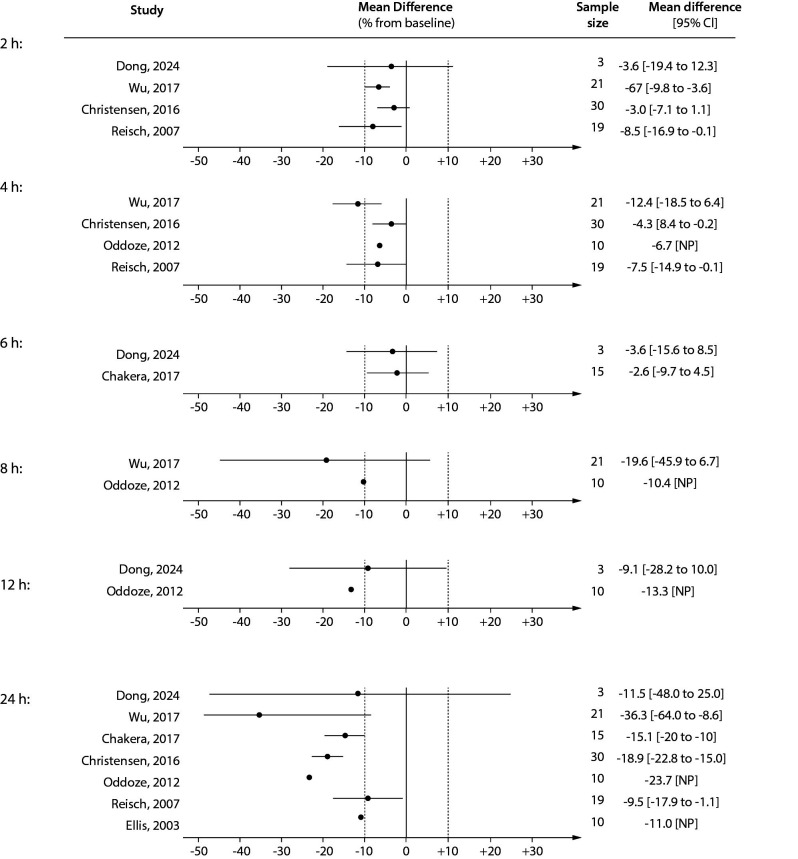
Forest plots depicting the stability of ACTH in uncentrifuged tubes at room temperature relative to the duration of storage prior to centrifugation. The X-axis represents the mean percentage deviation (PD%) compared to the baseline concentration. Bar errors correspond to 95% confidence intervals (CI). ACTH - adrenocorticotropic hormone. NP - not provided (indicating studies where the calculation of the 95% CI was not feasible with the available data, denoted by dots without error bars in the graphs).

Four studies provided data on the stability of ACTH concentration in uncentrifuged tubes stored at RT for a storage duration of 2 and 4 h, 2 studies for 6, 8, 12 h, and 7 studies for 24 h. All of these studies utilized collection tubes containing only EDTA. As illustrated in Figure 3, nearly all studies reported a mean PD% in ACTH concentrations relative to baseline below the 10% threshold when uncentrifuged tubes were stored at RT for 2, 4, and 6 h. The only exception was the study by Wu *et al.*, which reported a mean PD% of - 12.4% (95% CI: - 18.5 to 6.4) after 4 h of storage (*18*).

Nearly all studies investigating the stability of ACTH after 8, 12, and 24 h of storage at RT reported PD% greater than the 10% threshold.

## Discussion

Our systematic review of the literature argues that ACTH is stable in uncentrifuged EDTA tubes for 6 h at RT and at least 8 h under refrigerated conditions. Although we found that ACTH remains stable after a 12 h storage under refrigeration, this finding is drawn from only two studies, one of which did not provide a confidence interval for the PD%, while the other reported a wide confidence interval for the mean PD%. These limitations make it difficult to draw definitive conclusions regarding ACTH stability under this storage condition, and further stability studies are needed to conclude on the ACTH stability in whole blood for storage durations greater than 8 h under refrigerated conditions (*33*).

Our findings bring evidence that ACTH is less prone to degradation in whole blood than previously assumed. In 2002, the World Health Organization reported on the instability of ACTH in whole blood at RT, but without specifying the duration of stability (*34*). In 2010, the German Society for Clinical Chemistry and Laboratory Medicine suggested stability in whole blood for a large range from 1 to 4 h at RT, but without providing recommendations for the refrigerated condition (*35*). However, the conclusions of the two latter reports were based on the review of only 3 and 4 reports, respectively, whereas our systematic review encompassed data from 9 studies, all conducted since 2010.

Our work has also limitations that warrant discussion. Firstly, our search strategy was limited to the PubMed database and to articles published in English. Therefore, it is possible that we may have overlooked some eligible stability studies, although PubMed is one of the largest biomedical bibliographic databases. Secondly, the number of the selected studies was quite limited, making it statistically difficult to generate one pooled estimate in a meta-analysis and to study the possible moderators. Thirdly, our review did not permit to draw conclusions regarding the potential advantages of tubes containing both EDTA and protease inhibitors. Two studies provided data on the stability in such tubes, but we excluded these studies since they did not report directly the PD% (*29*, *30*). Casati *et al.* conducted a direct comparison between storage in EDTA *versus* EDTA-aprotinin tubes (*30*). They observed a higher decrease in ACTH concentrations with EDTA-aprotinin tubes compared to EDTA tubes after 2 h of storage in ice/water, but without statistical analysis of the difference (*30*). Unfortunately, no data were available for storage durations greater than 6 h at RT in EDTA-aprotinin tubes, for which ACTH degradation is unambiguously significant in EDTA tubes.

In summary, our study provides a comprehensive assessment of studies reporting on ACTH stability in uncentrifuged tubes. By demonstrating the stability of ACTH in uncentrifuged EDTA tubes for 6 h at RT and at least 8 h under refrigeration, our findings can assist clinical laboratories in reviewing their acceptance criteria for sample transport regarding time and temperature. Nonetheless, it is important to acknowledge that transport time and temperature constitute only one aspect of the conditions requiring careful management during the preanalytical phase (*36*). Other variables, such as recording the timing of blood sampling or hemolysis, deserve special attention for ACTH analysis (*5*).

## Data Availability

The data generated and analyzed in the presented study are available from the corresponding author on request.

## References

[1] Fleseriu M Auchus R Bancos I Ben-Shlomo A Bertherat J Biermasz NR Consensus on diagnosis and management of Cushing’s disease: A guideline update. Lancet Diabetes Endocrinol. 2021;9:847–75. 10.1016/S2213-8587(21)00235-7 34687601 PMC8743006

[2] Bornstein SR Allolio B Arlt W Barthel A Don-Wauchope A Hammer GD Diagnosis and treatment of primary adrenal insufficiency: An Endocrine Society Clinical practice guideline. J Clin Endocrinol Metab. 2016;101:364–89. 10.1210/jc.2015-1710 26760044 PMC4880116

[3] Owen CA. Leukocyte cell surface proteinases: regulation of expression, functions, and mechanisms of surface localization. Int J Biochem Cell Biol. 2008;40:1246–72. 10.1016/j.biocel.2008.01.020 18329945 PMC2425676

[4] Hillebrand JJ Zhou L Marcinkus MA Datwyler M Gawel SH Martens F Instability of corticotropin during long-term storage - myth or reality? Clin Chem Lab Med. 2021;60:60–5. 10.1515/cclm-2021-0818 34643074

[5] Lippi G Blanckaert N Bonini P Green S Kitchen S Palicka V Haemolysis: an overview of the leading cause of unsuitable specimens in clinical laboratories. Clin Chem Lab Med. 2008;46:764–72. 10.1515/CCLM.2008.170 18601596

[6] Bosse M Bayart JL. Crioplast is a reliable device to ensure pre-analytical stability of adrenocorticotrophin (ACTH). Clin Chem Lab Med. 2024;62:e123–5. 10.1515/cclm-2024-0043 38366955

[7] Dong L Hao T Chen J Chai Y Jia Z Tuo W Research on the stability changes in expert consensus of the ACTH detection preprocessing scheme. JLM. 2024;48:77–81. 10.1515/labmed-2023-0086

[8] Lippi G Simundic AM European Federation for Clinical Chemistry and Laboratory Medicine (EFLM) Working Group for Preanalytical Phase (WG-PRE). The EFLM strategy for harmonization of the preanalytical phase. Clin Chem Lab Med. 2018;56:1660–6. 10.1515/cclm-2017-0277 28593910

[9] Page MJ McKenzie JE Bossuyt PM Boutron I Hoffmann TC Mulrow CD The PRISMA 2020 statement: an updated guideline for reporting systematic reviews. BMJ. 2021;372(71): 10.1136/bmj.n71 33782057 PMC8005924

[10] Gómez Rioja R Martínez Espartosa D Segovia M Ibarz M Llopis MA Bauça JM Laboratory sample stability. Is it possible to define a consensus stability function? An example of five blood magnitudes. Clin Chem Lab Med. 2018;56:1806–18. 10.1515/cclm-2017-1189 29729140

[11] BD Vacutainer EDTA Tubes. Available from: https://www.bd.com/en-us/products-and-solutions/products/product-page.367863. Accessed Apr 1st 2024.

[12] Greiner Bio-One. Available from: https://www.gbo.com/. Accessed Apr 1st 2024.

[13] Sarstedt. Available from: https://www.sarstedt.com/fr/produits/diagnostic/sang-veineux/s-monovette/produit/01.1605.001/. Accessed Apr 29st 2024.

[14] Shi J Luo D Weng H Zeng XT Lin L Chu H Optimally estimating the sample standard deviation from the five-number summary. Res Synth Methods. 2020;11:641–54. 10.1002/jrsm.1429 32562361

[15] International Standardization Organisation. ISO 5725-6:1994. Available from: https://www.iso.org/standard/11837.html. Accessed Apr 1st 2024.

[16] Aarsand A, Fernandez-Calle P, Webster C. EFLM Biological Variation. Available from: https://biologicalvariation.eu/. Accessed Apr 1st 2024.

[17] Desirable Biological Variation Database specifications - Westgard. Available from: https://www.westgard.com/biodatabase1.htm. Accessed Apr 1st 2024.

[18] Wu ZQ Xu HG. Preanalytical stability of adrenocorticotropic hormone depends on both time to centrifugation and temperature. J Clin Lab Anal. 2017;31:e22081. 10.1002/jcla.22081 27735096 PMC6816861

[19] Jane Ellis M Livesey JH Evans MJ. Hormone stability in human whole blood. Clin Biochem. 2003;36:109–12. 10.1016/S0009-9120(02)00440-X 12633759

[20] Christensen M Madsen RF Møller LR Knudsen CS Samson MH. Whole blood samples for adrenocorticotrophic hormone measurement can be stored at room temperature for 4 hours. Scand J Clin Lab Invest. 2016;76:653–6. 10.1080/00365513.2016.1230887 27701894

[21] Reisch N Reincke M Bidlingmaier M. Preanalytical stability of adrenocorticotropic hormone depends on time to centrifugation rather than temperature. Clin Chem. 2007;53:358–9. 10.1373/clinchem.2006.080622 17259248

[22] Chakera AJ McDonald TJ Knight BA Vaidya B Jones AG. Current laboratory requirements for adrenocorticotropic hormone and renin/aldosterone sample handling are unnecessarily restrictive. Clin Med (Lond). 2017;17:18–21. 10.7861/clinmedicine.17-1-18 28148573 PMC6297593

[23] Turkon H Toprak B Yalcin H Colak A Ozturk N. The effectiveness of temperature versus aprotinin in maintaining the preanalytical stability of adrenocorticotrophin. Lab Med. 2016;47:279–82. 10.1093/labmed/lmw027 27593170

[24] Evans MJ Livesey JH Ellis MJ Yandle TG. Effect of anticoagulants and storage temperatures on stability of plasma and serum hormones. Clin Biochem. 2001;34:107–12. 10.1016/S0009-9120(01)00196-5 11311219

[25] Schneider S Brümmer V Carnahan H Dubrowski A Askew CD Strüder HK. Stress hormone stability: processing of blood samples collected during parabolic flight. A pre-flight comparison of different protocols. Clin Biochem. 2007;40:1332–5. 10.1016/j.clinbiochem.2007.07.022 17888897

[26] Wu ZQ Xu HG. Comparison of two commercial quality control sera for adrenocorticotropin (ACTH) used in Elecsys® immunoassay system. J Clin Lab Anal. 2019;33:e22618. 10.1002/jcla.22618 30006935 PMC6430340

[27] Hillebrand JJ Heijboer AC Endert E. Effects of repeated freeze-thaw cycles on endocrine parameters in plasma and serum. Ann Clin Biochem. 2017;54:289–92. 10.1177/0004563216657361 27303059

[28] Livesey JH Dolamore B. Stability of plasma adrenocorticotrophic hormone (ACTH): influence of hemolysis, rapid chilling, time, and the addition of a maleimide. Clin Biochem. 2010;43:1478–80. 10.1016/j.clinbiochem.2010.09.020 20875812

[29] Mechanic L Mendez A Merrill L Rogers J Layton M Todd D Planned variation in preanalytical conditions to evaluate biospecimen stability in the National Children’s Study (NCS). Clin Chem Lab Med. 2013;51:2287–94. 10.1515/cclm-2013-0454 23924524 PMC4100775

[30] Casati M Cappellani A Perlangeli V Ippolito S Pittalis S Romano R Adrenocorticotropic hormone stability in preanalytical phase depends on temperature and proteolytic enzyme inhibitor. Clin Chem Lab Med. 2013;51:e45–7. 10.1515/cclm-2012-0431 23023695

[31] Lambert A Frost J Ratcliffe WA Robertson WR. On the stability in vitro of bioactive human adrenocorticotrophin in blood and plasma. Clin Endocrinol (Oxf). 1985;23:253–61. 10.1111/j.1365-2265.1985.tb00221.x 3000649

[32] Diver MJ Hughes JG Hutton JL West CR Hipkin LJ. The long-term stability in whole blood of 14 commonly-requested hormone analytes. Ann Clin Biochem. 1994;31:561–5. 10.1177/000456329403100606 7880075

[33] Cornes M Simundic AM Cadamuro J Costelloe SJ Baird G Kristensen GBB The CRESS checklist for reporting stability studies: on behalf of the European Federation of Clinical Chemistry and Laboratory Medicine (EFLM) Working Group for the Preanalytical Phase (WG-PRE). Clin Chem Lab Med. 2020;59:59–69. 10.1515/cclm-2020-0061 32710715

[34] Technology WHODI and L. Use of anticoagulants in diagnostic laboratory investigations. 2002. Available from: https://iris.who.int/handle/10665/65957. Accessed Apr 1st 2024.

[35] Guder WG, da Fonseca-Wollheim F, Heil W, Schmitt Y, Töpfer G, Wisser H, et al. Quality of diagnostic samples. Recommendations of the working group on preanalytical quality of the German Society for Clinical Chemistry and Laboratory Medicine. Darmstadt: GIT, 2001.

[36] Vermeersch P Frans G von Meyer A Costelloe S Lippi G Simundic AM. How to meet ISO15189:2012 pre-analytical requirements in clinical laboratories? A consensus document by the EFLM WG-PRE. Clin Chem Lab Med. 2021;59:1047–61. 10.1515/cclm-2020-1859 33554545

[37] Nandakumar V Paul Theobald J Algeciras-Schimnich A. Evaluation of plasma ACTH stability using the Roche Elecsys immunoassay. Clin Biochem. 2020;81:59–62. 10.1016/j.clinbiochem.2020.04.004 32315613

[38] Oddoze C Lombard E Portugal H. Stability study of 81 analytes in human whole blood, in serum and in plasma. Clin Biochem. 2012;45:464–9. 10.1016/j.clinbiochem.2012.01.012 22285385

